# Establishment of an in‐house real‐time RT‐PCR assay for the detection of severe acute respiratory syndrome coronavirus 2 using the first World Health Organization international standard in a resource‐limited country

**DOI:** 10.1002/jcla.24355

**Published:** 2022-03-21

**Authors:** Linh Tung Nguyen, Phuong Minh Nguyen, Duc Viet Dinh, Hung Ngoc Pham, Lan Anh Thi Bui, Cuong Viet Vo, Ben Huu Nguyen, Hoan Duy Bui, Cuong Xuan Hoang, Nhat Minh Van Ngo, Truong Tien Dang, Anh Ngoc Do, Dung Dinh Vu, Linh Thuy Nguyen, Mai Ngoc Nguyen, Thu Hang Thi Dinh, Son Anh Ho, Luong Van Hoang, Su Xuan Hoang, Quyet Do

**Affiliations:** ^1^ 344759 Department of Occupational Medicine Vietnam Military Medical University Hanoi Vietnam; ^2^ 344759 Department of Epidemiology Vietnam Military Medical University Hanoi Vietnam; ^3^ Institute of Biomedicine Vietnam‐Russia Tropical Center Hanoi Vietnam; ^4^ 344759 Department of Human Anatomy Vietnam Military Medical University Hanoi Vietnam; ^5^ 344759 Department of Parasitology Vietnam Military Medical University Hanoi Vietnam; ^6^ 344759 Institute of Biomedicine and Pharmacy Vietnam Military Medical University Hanoi Vietnam; ^7^ Hanoi University of Science and Technology Hanoi Vietnam; ^8^ Faculty of Biology National University of Hanoi Hanoi Vietnam; ^9^ 344759 Military Hospital 103 Vietnam Military Medical University Hanoi Vietnam

**Keywords:** clinical performance, real‐time RT‐PCR, SARS‐CoV‐2

## Abstract

**Background:**

The COVID‐19 pandemic caused by SARS‐CoV‐2 remains public health burdens and many unresolved issues worldwide. Molecular assays based on real‐time RT‐PCR are critical for the detection of SARS‐CoV‐2 in clinical specimens from patients suspected of COVID‐19.

**Objective:**

We aimed to establish and validate an in‐house real‐time RT‐PCR for the detection of SARS‐CoV‐2.

**Methodology:**

Primers and probes sets in our in‐house real‐time RT‐PCR assay were designed in conserved regions of the *N* and *E* target genes. Optimized multiplex real‐time RT‐PCR assay was validated using the first WHO International Standard (NIBSC code: 20/146) and evaluated clinical performance.

**Results:**

The limit of detection validated using the first WHO International Standard was 159 IU/ml for both E and N target genes. The evaluation of clinical performance on 170 clinical samples showed a positive percent agreement of 100% and the negative percent agreement of 99.08% for both target genes. The Kappa value of 0.99 was an excellent agreement, the strong correlation of *C*_t_ values observed between two tests with *r* ^2^ = 0.84 for the E gene and 0.87 for the N gene. Notably, we assessed on 60 paired saliva and nasopharyngeal samples. The overall agreement was 91.66%, and Kappa value of 0.74 showed a high agreement between two types of samples. When using nasopharyngeal swabs as the reference standard, positive percent agreement, and negative percent agreement were 91.83% and 90.90%, respectively.

**Conclusion:**

In the present study, we established and validated an in‐house real‐time RT‐PCR for molecular detection of SARS‐CoV‐2 in a resource‐limited country.

## 1 INTRODUCTION

The outbreak of coronavirus disease 2019 (COVID‐19) pandemic caused by severe acute respiratory syndrome coronavirus 2 (SARS‐CoV‐2) remains a big challenge and public health burden in many countries associated with morbidity and mortality. According to the World Health Organization (WHO) report, COVID‐19 case incidence is still increasing in many regions on the world. Globally, there were nearly 265 million cases confirmed with SARS‐CoV‐2 infection and over 5.2 million deaths have been reported.^1^ Although several vaccines have been approved for emergency use authorization by the WHO and the US Food and Drug Administration (FDA), helped in preventing asymptomatic and symptomatic infections from SARS‐CoV‐2, and markedly reduced outcome of COVID‐19 in vaccinated individuals as compared to unvaccinated individuals^2^, ^3^; however, the emergence of novel SARS‐CoV‐2 variants has been shown to be associated with a rapid transmission of SARS‐CoV‐2 and breakthrough infection in fully vaccinated individuals.^4^, ^5^, ^6^ Real‐time RT‐PCR assay was considered as the gold standard for the diagnosis of SARS‐CoV‐2 infection in clinical specimens from suspected cases of COVID‐19.^7^ There were many commercial and laboratory‐developed real‐time RT‐PCR assays have become available for routine diagnosis of SARS‐CoV‐2 in clinical laboratories. However, some real‐time RT‐PCR kits failed for the detection of SARS‐CoV‐2 associated with the presence of mutations in primer and probe binding regions.^8^, ^9^, ^10^ Therefore, this study aimed to establish and validate an in‐house real‐time RT‐PCR for the detection of SARS‐CoV‐2 in resource‐limited settings.

## 2 MATERIALS AND METHODS

### 2.1 Primers and probes design

The complete sequences of SARS‐CoV‐2 were downloaded from the Genbank and GISAID database for alignment using BioEdit 7.0 (https://bioedit.software.informer.com/versions/) to select highly conserved regions of targets in the E and N genes for design primers and probes with the aid of Primer Express software version 3.0 (Thermo Fisher Scientific). The primers and probe set of N target are specific to SARS‐CoV‐2, whereas E gene primers and probe will detect both SARS‐CoV and SARS‐CoV‐2 but not detect other coronaviruses. In addition, we used primers and probe set of Rase P published by USA‐CDC as an internal control in our assay.^11^ Primers and probes sequences are summarized in Table 1. Primers and probes were purchased from IDT.

**TABLE 1:** Primers and probes sequences used in this study

Primers and probes	Sequences
VE1‐Pr	TEXAS‐RED‐5′‐AACCGACGACGACTACTAGCGTGCCTT‐3′‐BHQ1
VE6‐F	5′‐CGGAGTTGTTAATCCAGTAATGGA‐3′
VE6‐R	5′‐GTTCGTACTCATCAGCTTGTGCTT‐3′
qVN‐F	5′‐GGTCCAGAACAAACCCAAGGA‐3′
qVN‐R	5′‐GACATTCCGAAGAACGCTGAA‐3′
qVN‐Pr	FAM‐5′‐ATTGCACAATTTGCCCCCAGCG‐3′‐BHQ1
Rnase P‐F	5′‐AGATTTGGACCTGCGAGCG‐3′
Rnase P‐R	5′‐GAGCGGCTGTCTCCACAAGT‐3′
Rnase P‐Pr	HEX‐TTCTGACCTGAAGGCTCTGCGCG‐3′‐BHQ1
S‐Fm	5′‐AGGGCAAACTGGAAAGATTGCT‐3′
S‐Rm	5′‐CAGCCCCTATTAAACAGCCTGC‐3′
N‐Fs	5′‐ACAACAAGGCCAAACTGTCAC‐3′
N‐Rs	5′‐TGTCTCTGCGGTAAGGCTTG‐3′

### 2.2 Real‐time RT‐PCR:

We optimized a multiplex real‐time RT‐PCR assay (designated as Laboratory Developed Assay: LDA assay) in a total volume of 20 µl containing 10 µl of 2X Luna^®^ Universal probe one‐step RT‐qPCR Kit (New England Biolab), 1 µl of RT enzyme, 1 µl each of 10 µM forward and reverse primers of N and E genes, and the 0.4 µl each of 5 µM probes of N and E genes, 0.8 µl each of 10 µM forward and reverse primers of Rnase P and 0.4 µl of 5 µM Rnase P probes and 5 µl of RNA template. All reactions were run on a RotorGene Q 5plex MDx (Qiagen) using the following thermal cycling conditions: 50°C for 2 min, followed by 45 cycles of 90° C for 15 min, 94° C for 15 s, and 58°C for 60 s.

### 2.3 LiliF COVID‐19 real‐time PCR kit

LiliF COVID‐19 real‐time RT‐PCR (iNtRON Biotechnology) used in this study as the reference assay to validate our established in‐house LDA assay on clinical samples. This kit was designed for the detection of SARS‐CoV‐2 using three target genes: envelope (E), RNA‐dependent RNA polymerase (RdRp), nucleocapsid (N), and Rnase P as internal control. According to the manufacturer's interpretation, diagnosis of SARS‐CoV‐2 is confirmed for a sample that has at least three genes with cycle threshold (*C*_t_)‐value ≤35.

### 2.4 Validation of real‐time RT‐PCR using the first WHO International standard

The first WHO International Standard for SARS‐CoV‐2 RNA for Nucleic acid Amplification Technique (NAT)‐based assays (NIBSC code: 20/146) was provided as a kind gift from Dr. Do Minh Si, Nanogen Biopharma, Vietnam. This material was reconstituted in 0.5 ml of molecular grade water to obtain the final concentration of 7.70 Log10 IU/ml as recommended by the manufacturer. This material was extracted using Qiagen RNA viral mini kit (Qiagen) according to instruction and a serial dilution of standards from 5.02 × 10^5^ to 5.02 IU/ml were prepared in Ambion RNA storage solution (catalog number AM7001; Thermo Fisher Scientific) using for validation of our assay. The limit of detection (LOD) was defined at the lowest concentration that can be detected with the probability of 95% using probit analysis. For analytical specificity, we used the coronavirus RNA specificity panel obtained by the European virus archive global (EVAg), https://www.european‐virus‐archive.com and other pathogens stored in our laboratory.

### 2.5 Clinical specimens

Nasopharyngeal specimens were collected from patients suspected of COVID‐19 admitted into the Field hospital deployed in Bac Giang province in the third wave of COVID‐19 outbreak from May to June 2021 in Vietnam. Ethical approval was obtained from the local authorities for all samples of the study. A total of 170 nasopharyngeal swabs in viral transport medium (VTM) used for the comparative evaluation of clinical performance. Of these samples, 62 nasopharyngeal swabs were collected from patients confirmed by a positive nasopharyngeal swab at admission of field hospital, whereas 108 samples were negative from individuals at risk in contact tracing using the Corman's E gene primer/probe set validated in our laboratory,^12^ whereas CDC's N2 gene primer/probe used for confirmatory detection of SARS‐CoV‐2 as protocol published by Lu et al.^11^ In another cohort, 50 pairs of saliva–nasopharyngeal swabs were collected simultaneously within the first week from patients of COVID‐19, and 10 negative paired samples were randomly selected for comparative evaluation of our LDA assay. The viral RNA was extracted from 140 μl of nasopharyngeal swabs in 3 ml of VTM and saliva samples collected in a sterile nuclease‐free falcon tube using QIAamp Viral RNA Mini Kit (Qiagen GmbH) according to the manufacturer's instruction. The RNA was finally eluted in a final volume of 60 μl of AE buffer and was stored at −70°C until use.

#### 2.5.1 Sequencing confirmation and phylogenetic analysis

To assess the ability of the proposed method to amplify the SARS‐CoV‐2 variants. A 725 bp fragment of Spike gene was amplified by one‐step RT‐PCR (Qiagen GmbH) from samples detected positive by our method using primer sequences previously described.^13^ In addition, we designed primers to amplify a 438‐bp fragment covering primers and probe sequence region of N target gene. Amplified fragments were visualized under UV light, and then purified and sequenced using a 3130 XL sequencer. Sequences obtained were aligned with reference sequences retrieved from GenBank and GISAID (Table 7) using Bioedit 7.0 (https://bioedit.software.informer.com/versions/) and MEGA 7.0 software (www.megasoftware.net). Phylogenetic tree was constructed using the neighbor‐joining method, and significance level was estimated with 1000 bootstrap replicates.

### 2.6 Statistical analyses

Statistical analyses were done with SPSS 20.0 (IBM). The diagnostic agreements were analyzed to estimate confidence intervals (95% CI) for positive percent agreement (PPA) and negative percent agreement (NPA). Cohen's kappa values were calculated for evaluating overall agreement and comparing assays. Correlation analysis was used to evaluate the *C*_t_ values of the positive results. *p* values <0.05 were considered as statistically significant.

## 3 RESULTS

### 3.1 Validation of analytical sensitivity and specificity of real‐time RT‐PCR

#### 3.1.1 Analytical sensitivity

A serial dilution of standards as prepared above and real‐time RT‐PCR reactions were run in eight replicates of each dilution. Results were presented Table 2. By using probit analysis, the LOD determined for both targets were 159 IU/ml.

**TABLE 2:** Limits of detection of real‐time RT‐PCR for detection of SARS‐CoV‐2 using the 1st WHO international standard (NIBSC code: 20/146)

Target genes	E		N	
Conc (IU/ml)	Detected	Replicates	Detected	Replicates
50,200	8	8	8	8
5020	8	8	8	8
502	8	8	8	8
251	8	8	8	8
50.2	6	8	6	8
25.1	7	8	7	8
5.02	0	8	0	8

Abbreviations: Conc, concentratio; IU, international unit.

The reproducibility of real‐time RT‐PCR assay was assessed by coefficient of variation (CV) of the cycle threshold (*C*_t_) values in the intra‐ and inter‐assays at three different concentrations using the first WHO International Standard (5.02 × 10^5^ IU/ml, 5.02 × 10^4^ IU/ml, 5.02 × 10^3^ IU/ml). For intra‐assay repeatability, each concentration was tested triplicate in one reaction, for inter‐assay reproducibility, each concentration was run in three independent reactions across three different days. The results are presented in Table 3. The mean CV of *C*_t_ values was observed to be lesser 5% for both concentrations evaluated, found an accurate and a good repeatability of our LDA assay.

**TABLE 3:** Intra‐assay and inter‐assay reproducibility

	Concentration (IU/ml)	gene N			gene E		
		Mean *C*_t_	SD	CV (%)	Mean *C*_t_	SD	CV (%)
Inter‐assay	5.02E+05	24.68	0.21	0.85	26.13	0.66	2.52
	5.02E+04	28.26	0.88	3.11	29.59	1.23	4.14
	5.02E+03	31.61	0.35	1.11	33.35	0.64	1.92
Intra‐assay	5.02E+05	24.52	0.21	0.84	25.40	0.04	0.16
	5.02E+04	27.92	0.14	0.49	28.74	0.23	0.79
	5.02E+03	31.43	0.39	1.25	32.75	0.11	0.34

Abbreviations: *C*_t_, cycle threshold; CV, coefficient of variation; IU, international unit; SD, standard deviation.

#### 3.1.2 Analytical specificity

To evaluate the specificity of our LDA assay, we tested on the coronavirus RNA specificity panel obtained by the European virus archive global (EVAg), https://www.european‐virus‐archive.com, included four human *coronaviruses* (MERS‐CoV, HCOV‐OC43, HCOV‐229E, and HCOV‐NL63). There was no cross‐reactivity with other coronaviruses tested.

### 3.2 Evaluation of clinical performance

**FIGURE 1:**
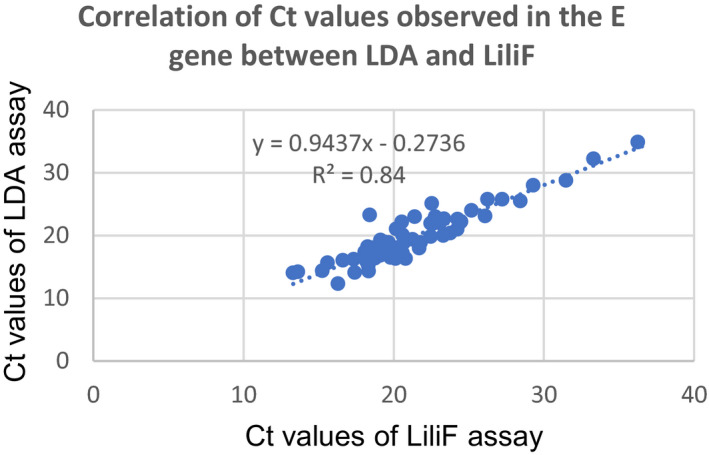
Correlation analysis for the *C*_t_ values of E gene between LDA and LiliF assay

**FIGURE 2:**
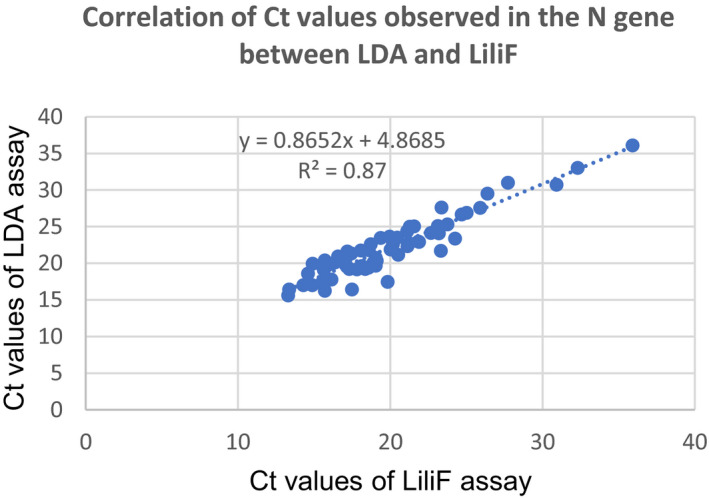
Correlation analysis for the *C*_t_ values of N gene between LDA and LiliF assay

A total of 170 nasopharyngeal swabs collected from patients suspected of COVID‐19 were tested previously with validated protocols using Corman's E gene primer/probe set and USA‐CDC's N2 gene primer/probe set detected 62 samples positive with the *C*_t_ values range from 15.25 to 34.84 and 14.39 to 36.93 for E and N2 gene, respectively, whereas 108 samples were negative. To evaluate clinical performance of our LDA assay, we compared with a commercial LiliF COVID‐19 real‐time RT‐PCR (now designated as LiliF assay) as reference assay, there were 62 samples detected positive with both the E and N target genes in our LDA assay, whereas only 61 samples tested positive with the E and N target gene of LiliF assay, indicating a positive percent agreement of LDA assay was 100% and Kappa value of 0.99 (95% CI, 0.96–1.00) showed an excellent agreement. While the NPA was 99.08% for both target genes (Table 4). A sample was positive in our LDA assay with *C*_t_ values of E and N gene were 36 and 36.1, respectively, but negative with LiliF assay. This discordant sample was retested with LiliF assay remains negative. In addition, we showed a strong correlation of *C*_t_ values observed between two tests with *r* ^2^ = 0.84 (Figure 1) for the E gene and 0.87 for the N gene (Figure 2).

**TABLE 4:** Clinical performance of our LDA assay and reference assay on nasopharyngeal swabs

SARS‐CoV‐2 assay		Reference assay		Kappa	PPA (95% CI)	NPA (95% CI)
		Positive	Negative	(95% CI)		
		E‐LiliF				
E‐LDA	Positive	61	1	0.99	100%	99.08%
	Negative	0	108	(0.96–1.00)	(94.13–100%)	(94.99–99.98%)
		N‐LiliF				
N‐LDA	Positive	61	1	0.99	100%	99.08%
	Negative	0	108	(0.96–1.00)	(94.13–100%)	(94.99–99.98%)

Abbreviations: CI, confident interval; LDA, laboratory‐developed assay; NPA, negative percent agreement; PPA, positive percent agreement.

**FIGURE 3:**
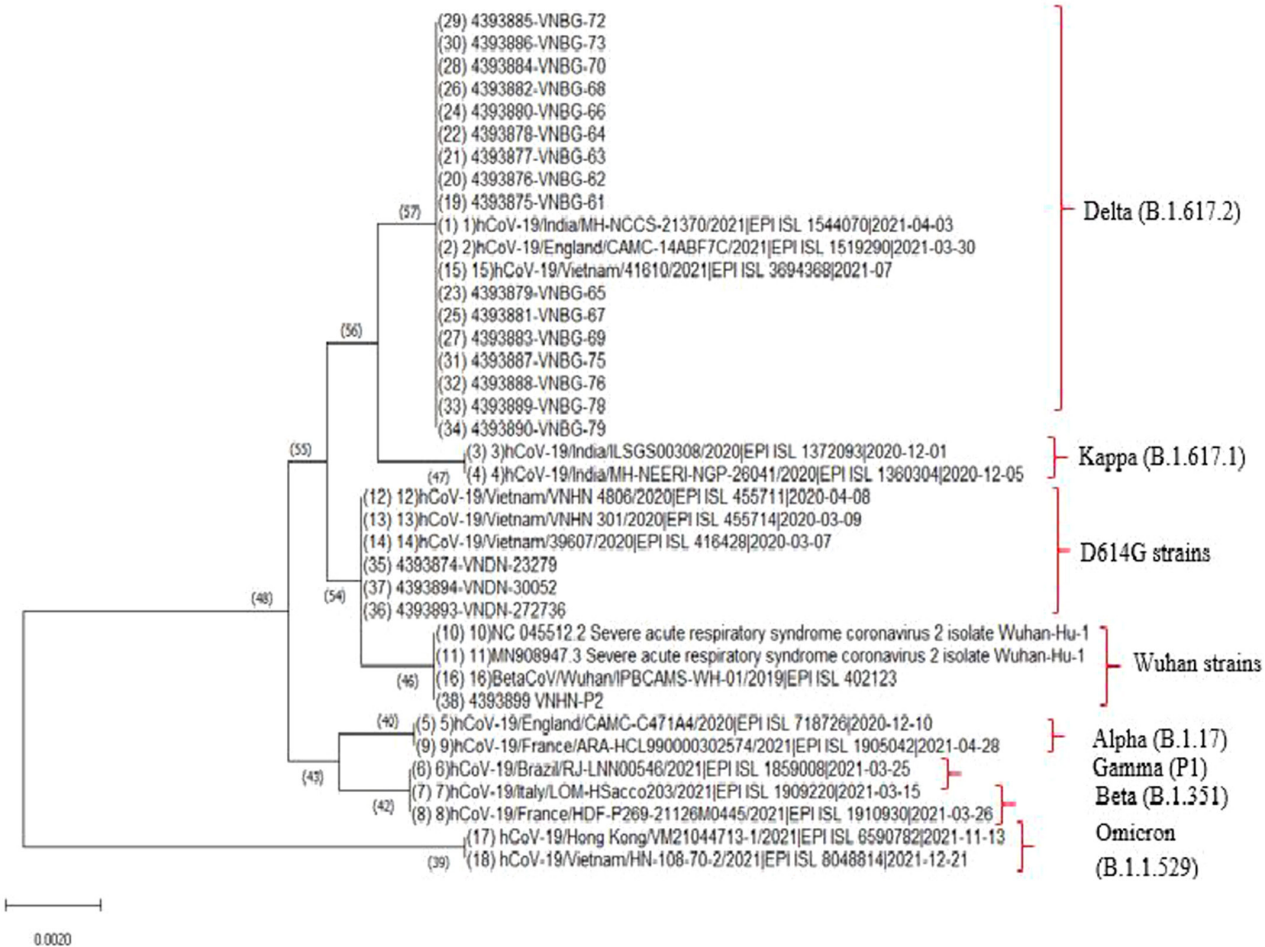
Phylogenetic tree was constructed using the Spike partial gene sequences (22,798–23,522) obtained in this study, and 18 reference sequences of SARS‐CoV‐2 Wuhan strains retrieved from Genbank and GISAID (MN908947.3, NC_045512.2, EPI_ISL_402123, EPI_ISL_416428, EPI_ISL_455714, and EPI_ISL_455711) and SARS‐CoV‐2 Alpha (EPI ISL 718726 and EPI ISL 1905042), Beta (EPI ISL 1910930 and EPI ISL 1909220), Gamma (EPI ISL 1859008), Kappa (EPI ISL 1360304 and EPI ISL 1372093), Delta (EPI_ISL_3694368, EPI ISL 1544070, and EPI ISL 1519290), Omicron (EPI ISL 8048814 and EPI ISL 6590782) variants using CLUSTAL_W with Kimura's correction

**FIGURE 4:**
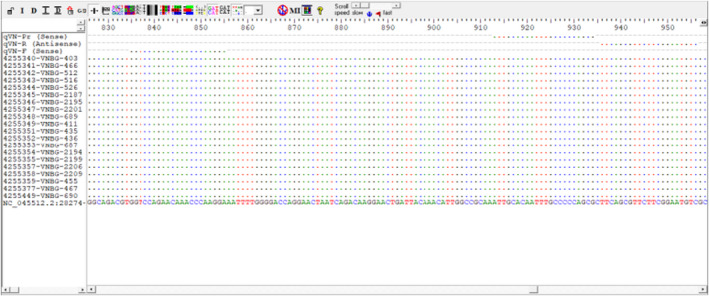
Alignment of primers (qVN‐F and qVN‐R) and probe (qVN‐Pr) of our LDA assay with sequence data of N gene obtained in this study and reference sequence (NC_045512.2) retrieved from GenBank

Furthermore, we evaluated on 50 paired saliva and nasopharyngeal samples from patients of COVID‐19, and 10 negative paired samples were randomly collected. Among 50 samples detected positive with the *C*_t_ values range from 15.85 to 35.27 and 14.01 to 34.24 for E and N gene, respectively, 45 samples had concordant results in saliva and nasopharyngeal swab, there were four samples detected SARS‐CoV‐2 in nasopharyngeal swab but not in saliva and only one case was positive in saliva but negative in nasopharyngeal swab. The overall agreement between two types of samples was 91.66% (55/60). When using nasopharyngeal swabs as the reference standard, positive percent agreement and NPA were 91.83% and 90.9% for both target genes, respectively. There was a high agreement (Kappa value = 0.74) between the two types of specimens (Table 5). However, the mean *C*_t_ values of nasopharyngeal swabs were significantly lower than that of saliva for both E and N target genes. Detailed data were presented in Table 6. This finding may be using saliva as an alternative specimen for mass screening of SARS‐CoV‐2 at the early stage of infection and epidemiological studies or surveillance of COVID‐19. To assess the impact of SARS‐CoV‐2 variants on our LDA, we analyzed in silico primers and probes designed in our in‐house LDA assay by mapping with the sequences of SARS‐CoV‐2 variants retrieved from GISAID. Sequences of SARS‐CoV‐2 variants are summarized in Table 7. There were no mismatches observed in primers and probes binding regions of E and N target genes. In addition, we sequenced 20 positive samples using primers designed to amplify a fragment covering N target region of our LDA assay. Sequence analysis obtained showed 100% nucleotide identity to primers and probe sequences of N target gene (Figure 4). On the contrary, 20 samples detected positive by our LDA assay were sequenced for Spike partial gene and compared with the reference sequence (NC_045512.2), identified three key mutations at L452R, T478K, and D614G in part of Spike gene associated with Delta variant in 16 out of 20 samples. For the phylogenetic analysis, 20 SARS‐CoV‐2 sequences obtained in this study were aligned with 18 reference sequences retrieved from the GISAID and GenBank using Bioedit 7.0 and MEGA 10.0 software. The results detected in 16 sequences were clustered into Delta variant branch (B.1.617.2 lineage), three sequences belonged to SARS‐CoV‐2 D614G strains, and 1 sequence was Wuhan Hu‐1 strains (Figure 3).

**TABLE 5:** Clinical performance of our LDA assay on nasopharyngeal swabs versus saliva specimens

Saliva specimens		Nasopharyngeal swabs		Kappa	PPA (95% CI)	NPA (95% CI)
		Positive	Negative	95% CI		
Gene‐E	Positive	45	1	0.74	91.83%	90.90%
	Negative	4	10	0.50–0.93	80.39–97.73%	58.72–99.77
Gene‐N	Positive	45	1	0.74	91.83%	90.90%
	Negative	4	10	0.52–0.94	80.39–97.73%	58.72–99.77

Abbreviations: CI, confident interval; NPA, negative percent agreement; PPA, positive percent agreement.

**TABLE 6:** Comparison of mean *C*_t_ values of LDA assay between nasopharyngeal swabs and saliva specimens

Assay	Target genes	Nasopharyngeal swabs	Saliva	*p* values
		Mean ± SD		
LDA	Gene‐N	21.23 ± 4.97	24.79 ± 6.10	0.002
	Gene‐E	23.54 ± 4.77	26.02 ± 5.32	0.019

Abbreviations: LDA, laboratory‐developed assay; SD: standard deviation.

**TABLE 7:** Reference sequences used in this study

Access. No	Strains	Reference source	Countries
MN908947.3	SARS‐CoV‐2 Wuhan Hu‐1	Genbank	China
NC045512.2	SARS‐CoV‐2 Wuhan Hu‐1	Genbank	China
AY508724.1	SARS‐CoV	Genbank	China
MG772934.1	bat‐SARS like *coronavirus*	Genbank	China
MN985325.1	SARS‐CoV‐2 Wuhan Hu‐1	Genbank	United States
MN988713.1	SARS‐CoV‐2 Wuhan Hu‐1	Genbank	United States
JX869059.2	Human beta coronavirus	Genbank	Netherlands
NC005831.2	Human Coronavirus NL63	Genbank	Netherlands
NC002645.1	Human Coronavirus 229E	Genbank	Germany
AY391777.1	Human Coronavirus OC43	Genbank	Belgium
NC006577.2	Human Coronavirus HKU1	Genbank	Hong Kong, China
EPI_ISL_402119	SARS‐CoV‐2 Wuhan Hu‐1	GISAID	China
EPI_ISL_402120	SARS‐CoV‐2 Wuhan Hu‐1	GISAID	China
EPI_ISL_402128	SARS‐CoV‐2 Wuhan Hu‐1	GISAID	China
EPI_ISL_403962	SARS‐CoV‐2 Wuhan Hu‐1	GISAID	Thailand
EPI_ISL_404228	SARS‐CoV‐2 Wuhan Hu‐1	GISAID	China
EPI_ISL_406844	SARS‐CoV‐2 Wuhan Hu‐1	GISAID	Australia
EPI_ISL_406596	SARS‐CoV‐2 Wuhan Hu‐1	GISAID	France
EPI_ISL_406597	SARS‐CoV‐2 Wuhan Hu‐1	GISAID	France
EPI_ISL_3694262	Delta (B.1.617.2)	GISAID	Vietnam
EPI_ISL_3694266	Delta (B.1.617.2)	GISAID	Vietnam
EPI_ISL_3694268	Delta (B.1.617.2)	GISAID	Vietnam
EPI_ISL_402123	SARS‐CoV‐2 Wuhan Hu‐1	GISAID	China
EPI_ISL_416428	SARS‐CoV‐2‐D614G	GISAID	Vietnam
EPI_ISL_455714	SARS‐CoV‐2‐D614G	GISAID	Vietnam
EPI_ISL_455711	SARS‐CoV‐2‐D614G	GISAID	Vietnam
EPI_ISL_3694368	SARS‐CoV‐2 Delta (B.1.617.2)	GISAID	Vietnam
EPI ISL 1544070	SARS‐CoV‐2 Delta (B.1.617.2)	GISAID	India
EPI ISL 1519290	SARS‐CoV‐2 Delta (B.1.617.2)	GISAID	England
EPI ISL 1360304	SARS‐CoV‐2 Kappa (B.1.617.1)	GISAID	India
EPI ISL 1372093	SARS‐CoV‐2 Kappa (B.1.617.1)	GISAID	India
EPI ISL 1905042	SARS‐CoV‐2 Alpha (B.1.17)	GISAID	France
EPI ISL 718726	SARS‐CoV‐2 Alpha (B.1.17)	GISAID	England
EPI ISL 1859008	SARS‐CoV‐2 Gamma (P1)	GISAID	Brazil
EPI ISL 1910930	SARS‐CoV‐2 Beta (B.1.351)	GISAID	France
EPI ISL 1909220	SARS‐CoV‐2 Beta (B.1.351)	GISAID	Italy
EPI ISL 8048814	SARS‐CoV‐2 Omicron (B.1.1.529)	GISAID	Vietnam
EPI ISL 6590782	SARS‐CoV‐2 Omicron (B.1.1.529)	GISAID	Hong Kong, China

## 4 DISCUSSIONS

COVID‐19 pandemic posed significant burdens for healthcare systems and interrupted many global socioeconomic issues. In response to the outbreak of COVID‐19 pandemic, there were many academic institutions and manufacturers developed and evaluated molecular methods for the detection of SARS‐CoV‐2.^14^ However, the emergence of new SARS‐CoV‐2 variants with many harboring point mutations may affect the sensitivity of diagnostic assays or lead to false‐negative results.^15^, ^16^, ^17^ Therefore, it is very crucial to select highly conserved regions for design primers and probes of real‐time RT‐PCR assays for accurately detect SARS‐CoV‐2 RNA in clinical specimens. Although there were many commercial molecular diagnostic assays, but it required modern equipment, high cost, and technical expertise. In this study, we developed and validated an affordable in‐house molecular assay using the first WHO International Standard (NIBSC code: 20/146) for the detection of SARS‐CoV‐2. To avoid underdiagnoses or false‐negative results due to single nucleotide polymorphisms, indel mutations occurred in primers and probes binding regions of diagnostic assays, we designed primers and probe targeting E gene for screening both SARS‐CoV‐2 and other beta coronaviruses, while primers and probe targeting N gene were designed specific for the detection of SARS‐CoV‐2. Then, optimized multiplex real‐time RT‐PCR assay was validated using the first WHO International Standard with a series of dilutions from 5.02 × 10^5^ to 5.02 IU/ml, showed a limit of detection was 159 IU/ml for both target genes, which is comparable with other available assays. The design strategy with at least two target sequences were amplified in a single tube of real‐time RT‐PCR reaction, which was widely used by many manufacturers and laboratory‐developed tests instead of using each target gene in diagnostic assay at the beginning of COVID‐19 outbreak.^18^, ^19^, ^20^, ^21^ Additionally, E and N target genes have been reported to be more sensitive, while *RdRp* was lower sensitivity and *S* gene had many mutations identified and may result in failed amplification of target sequence in molecular diagnosis.^22^, ^23^, ^24^

For clinical performance, the our LDA assay had an equivalent diagnostic performance as compared to a commercial LiliF assay on 170 nasopharyngeal swabs. The PPA was 100%, and NPA was 99% for both E and N target genes. The Kappa value was 0.99, showed a perfect agreement between two tests. However, there was a discordant result that was detected positive in our LDA with *C*_t_ values of E and N gene were 36 and 36.1, respectively, but tested negative by LiliF assay. In fact, diagnosis criteria of LiliF assay for the detection of SARS‐CoV‐2 RNA in clinical specimens were set with a *C*_t_ value of <35; therefore, it could not detect SARS‐CoV‐2 RNA for a sample had a low viral load, corresponding with a high *C*_t_ value of real‐time RT‐PCR assay.^17^ Interestingly, among positive samples, a strong correlation between the *C*_t_ values of E and N genes observed between our LDA and LiliF (Figures 1 and 2). Our findings were similar with previous reports as LDA assays were compared with commercial molecular tests or WHO's protocol and modified CDC's panel.^25^, ^26^ Interestingly, no mismatches observed in primers and probes binding regions as compared to reference sequences of SARS‐CoV‐2 variants of concern used this study. Sequencing data of positive samples confirmed the accuracy of our LDA assay. Further analysis, we evaluated on paired saliva–nasopharyngeal swab showed a significant agreement between two types of samples and using nasopharyngeal swab as the gold standard indicated a high PPA of 91.83% and the significant difference observed as compared mean *C*_t_ values of two sample types (Table 6). These study findings showed the similar sensitivity of saliva‐based real‐time RT‐PCR compared with paired positive nasopharyngeal (NP) samples ranging from 84% to 100% has been reported by other authors.^27^ Notably, these saliva specimens may be self‐collection or outside of hospital setting without assisting of nurse and saliva collection was a noninvasive procedure, avoiding a discomfort to the patients. However, quality of self‐collected saliva can mix sputum, mucus resulted in decreased sensitivity or inhibited PCR reactions. Therefore, saliva test may be suitable for high‐endemic regions, low incoming countries, and community surveillance, at the early stage of infection in clinical settings meanwhile nasopharyngeal swabs are critical for confirmation of disease cases of COVID‐19, follow‐up and making a decision for discharging patients from isolation.

This study result highlighted the benefit of an in–house real‐time RT‐PCR assay was standardized and validated with the WHO reference standards in response to urgent testing capacity and situation of global shortage of supply chain.

In conclusion, the present study, we developed and evaluated an affordable in‐house real‐time RT‐PCR assay for the detection of SARS‐CoV‐2 in clinical specimens in patients suspected of COVID‐19. This helps to benefit an affordable LDA assay for the effective control of COVID‐19 in resource‐limited settings.

## CONFLICT OF INTEREST

The authors have no conflicts of interest to disclose.

## AUTHOR CONTRIBUTIONS

N.T.L, N.M.P, D.V.D, and H.X.S conceived and designed, supervised study, drafted the article, and critically revised the article. B.T.L.A, B.D.H, N.V.N.M, D.N.A, N.V.B, N.T.L, N.N.M, and V.D.D performed experiments and contributed to acquisition of data, analysis, and interpretation of data. V.V.C, D.T.T, D.T.T.H, and H.A.S contributed to material support, acquisition of data, and commented on the article. P.N.H, H.X.C, H.V.L, D.Q critically revised the article.

## Data Availability

All data in this study can be obtained directly from the corresponding author.
